# Ten Years of Positive Impact of a Conservation Education Program on Children's Knowledge and Behaviour Toward Crested Macaques (*Macaca nigra*) in the Greater Tangkoko Area, North Sulawesi, Indonesia

**DOI:** 10.1007/s10764-023-00356-9

**Published:** 2023-05-03

**Authors:** Mathilde Chanvin, François Lamarque, Nona Diko, Muhammad Agil, Jérôme Micheletta, Anja Widdig

**Affiliations:** 1 Macaca Nigra Project, Tangkoko Reserve, Bitung, Indonesia; 2 Faculty of Veterinary Medicine, Bogor Agricultural University, Bogor, Indonesia; 3 Centre for Comparative and Evolutionary Psychology, Department of Psychology, University of Portsmouth, Portsmouth, UK; 4 Department of Human Behaviour, Ecology and Culture, Max Planck Institute for Evolutionary Anthropology, Deutscher Platz 6, 04103 Leipzig, Germany; 5 Behavioural Ecology Research Group, Institute of Biology, University of Leipzig, Leipzig, Germany; 6 German Centre for Integrative Biodiversity Research (iDiv) Halle-Jena-Leipzig, Leipzig, Germany

**Keywords:** Conservation education, Evaluation, Questionnaires, Tangkoko Conservation Education, Crested macaques, *Macaca nigra*, Sulawesi, Indonesia

## Abstract

In areas where primates are threatened, environmental education interventions are a key way to increase the local population’s knowledge of their environment and encourage positive attitudes and habits to preserve the environment and wildlife on a local and global scale. This study assesses the impact of Tangkoko Conservation Education (TCE), the Macaca Nigra Project’s conservation education programme, running since 2011 in North Sulawesi for school children, teachers, and the local population. TCE’s goal is to help people increase their knowledge of their environment and develop more positive habits and behaviours towards their local environment, especially the Critically Endangered and endemic crested macaques (*Macaca nigra*). We measured the efficacy of TCE’s programme for pupils using questionnaires provided one month before and one month after a year-long conservation education programme at school. Pupils’ knowledge and behaviour scores increased significantly after their participation in the programme. Their habits score also increased but this increase was not statistically significant. Girls scored significantly higher than boys in terms of positive behaviour towards wildlife. Children participating in the programme more than once seemed to obtain higher scores in their second participation, although the sample size was too small for formal analysis. Despite some limitations, this study demonstrates the positive impact of our programme on young people living in an area where primates are threatened. We hope that this research will inspire similar programmes in Sulawesi and elsewhere by providing methods and activities to help prevent primate extinction.

**Supplementary Information:**

The online version contains supplementary material available at 10.1007/s10764-023-00356-9.

“In the end we will conserve only what we love, we will love only what we understand, and we will understand only what we are taught” (Baba Dioum, speech delivered at the General Assembly of the IUCN, New Delhi, 1968).

## Introduction

Conservation education, the process by which individuals are taught ways to appreciate and protect the environment (Sherrow, 2010), is considered as one of the crucial strategies of wildlife conservation projects (Jacobson, 2010; Wallis & Lonsdorf, 2010; Whittaker, 2006). Where primates are threatened, environmental education interventions are a key way to increase local population’s awareness of and familiarity with local species (i.e., their knowledge). This can encourage positive emotions and beliefs (i.e., attitudes) as well as concrete actions (i.e., behaviours) in favour of these species and wildlife more generally (Bernárdez-Rodriguez *et al*., 2021; Freund *et al*., 2020; Kling & Hopkins, 2015), favouring the preservation of the environment on a local and global scale (Stevenson, 2007). To use limited governmental, charitable, and individual resources wisely, we need to assess the efficacy with which these educational interventions influence human behaviour and their impact on the environment (Bettinger *et al*., 2010; Fahlquist, 2009). The literature suggests that not all environmental education programmes evaluate their methods and especially the impact on their participants (Ardoin *et al*., 2020; Bernárdez-Rodriguez *et al*., 2021; Bettinger *et al*., 2010; Flowers, 2010; Kuhar *et al*., 2010; Linder *et al*., 2022; Wallis & Lonsdorf, 2010). In a review of 43 environmental education programmes focusing on primate conservation, 30% did not evaluate their methods and impact (Kling & Hopkins, 2015). Furthermore, most published evaluations of primate conservation education programmes focus on great apes’ conservation (i.e., chimpanzees, orangutans, gorillas, bonobos), possibly because they are seen as more charismatic species by the western public, compared with other primate families (Albert *et al*., 2018; Riley Koenig *et al*., 2019).

Of primate conservation education programmes that do evaluate their methods, most use surveys provided to participants before and immediately after their participation in the programme (Ballouard *et al*., 2011; Borchers *et al*., 2014; Kling & Hopkins, 2015; Savage *et al*., 2010). Most studies that report outcomes find positive results in terms of knowledge increase, which contributes to positive change in behaviour and attitudes of participants (Borchers *et al*., 2014; Karimullah *et al*., 2022; Rakotomamonjy *et al*., 2015; Savage *et al*., 2010). For example, a survey that assessed potential conflicts between humans and nonhuman primates in Malaysia showed that higher education and better knowledge of nonhuman primates predicted more positive opinions and approaches to mitigate human-primate conflictual interactions (Karimullah *et al*., 2022). While this study shows the link between knowledge and behaviour, it does not specify whether the knowledge was preexisting or the result of environmental, education interventions. Nevertheless, such studies show how important knowledge increase is as a first step to change human behaviours and favour positive attitudes towards primates, especially because pupils who take part in primate conservation education programmes retain the information learnt several months and even years after their participation (Kuhar *et al*., 2010; Rakotomamonjy *et al*., 2015).

The evaluations of conservation education programmes also revealed the importance of several factors affecting proconservation attitudes. A review of gender differences in human-animal interactions showed that women have more positive attitudes towards animals and are more often involved in their protection (Herzog, 2007). Similar disparities in environmental attitudes and behaviours between genders exist regardless of age or geographical location (Zelezny *et al*., 2000) and may result from differences in empathic concerns between genders (Arnocky & Stroink, 2010). Participants from a higher socioeconomic background often show more positive attitudes towards wildlife (Karimullah *et al*., 2022) possibly because of a higher education level (Archer Engels & Jacobson, 2007).

Most conservation education programmes target their activities, and evaluate their impact, on young people and children for several reasons. First, people develop their attitudes towards the environment at an early age (Jacobson *et al*., 2006). Second, once children’s attitudes towards the environment are formed, there is evidence that they are more resistant to change compared with adults (Jacobson & McDuff, 1997). Third, teaching children is suggested to affect adults through intergenerational transfer (Rakotomamonjy *et al*., 2015; van de Wetering *et al*., 2022; Vaughan *et al*., 2003). Finally, today’s youth will be responsible for the protection of the remaining natural resources in the future (Borchers *et al*., 2014; Kling & Hopkins, 2015; Korhonen & Lappalainen, 2004).

In terms of teaching methods targeting young people, multidisciplinary and active educational methods and tools should be favoured, such as trips in the forest (Bettinger *et al*., 2010; Kuhar *et al*., 2010), other sensory and immersive activities in nature (Gigliotti, 1990), theatre, and nature clubs (Breuer & Mavinga, 2010) to help students to learn about biodiversity (Flowers, 2010; Jacobson, 2010; Kling & Hopkins, 2015). Yet, a recent survey showed that most approaches used are passive, meaning they are delivered through extracurricular lessons, workbooks, posters, or videos, which does not necessarily make students actively engage in nature activity nor make them really connected to their local environment (Kling & Hopkins, 2015). Repeated participation in conservation education program can facilitate information retention and promotes proconservation attitudes (Kruse & Card, 2004).

Sulawesi is part of the Wallacea region, one of the 25 biodiversity hotspots of the world for conservation priorities (Myers *et al*., 2000). Sulawesi is of particular interest with respect to primate evolution and endemism, because it hosts seven macaque species found nowhere else in the world (Riley, 2010; Supriatna *et al*., 2020). The crested macaque (*Macaca nigra*), only found in North Sulawesi, is the most Endangered of these species. It is listed as Critically Endangered (Lee *et al*., 2020) and is one of the world’s 25 most endangered primates (Danish *et al*., 2017) due to substantial habitat loss and hunting pressure, which have occurred for decades (O’Brien & Kinnaird, 1997; Rosenbaum *et al*., 1998; Sugardjito *et al*., 1989; Supriatna *et al*., 2020). The population has dramatically declined in recent decades, causing a loss of more than 75% of the original population (Palacios *et al*., 2012). The biggest population in its original distribution range can currently be found in the Tangkoko Nature Reserve in North Sulawesi, where approximately 2,000 crested macaques remain (Palacios *et al*., 2012). Although protected by Indonesian law, a conservation workshop identified overharvesting, illegal logging, land conversion, and lack of effective law enforcement and awareness as continued threats to the Tangkoko reserve and its inhabitants, including crested macaques (Hilser *et al*., 2013). Tackling these threats largely depends on the action of local and national authorities and the implementation of alternative strategies for economic development. However, one action that can easily be implemented together with the local population is environmental education programmes.

The Tangkoko Conservation Education programme (TCE) is an ongoing, long-term, conservation education programme established in the Greater Tangkoko area in 2011. TCE is the official education programme of the Macaca Nigra Project (MNP), a scientific research programme studying the biology, ecology, and conservation of crested macaques. TCE implements conservation education interventions for school pupils and awareness campaigns for local populations, mostly around the Tangkoko reserve (Municipality of Bitung), but also in other parts of North Sulawesi (city of Manado and North Minahasa Regency). The long-term perspective of this programme is to collaborate with the local population for them to become more familiar with their local environment and to understand the reasons behind environmental changes. Specifically, TCE works with the local population (i.e., local schools, municipalities, government bodies, local conservation NGOs, and nature clubs) to take appropriate conservation and sustainability action particularly with respect to the local wildlife, such as reducing the negative human impact on the local environment (e.g., illegal hunting and logging, having wildlife as pets). To achieve these objectives, TCE implements bimonthly conservation education activities at school for local pupils aged 5–17 years. The programme consists of seven original and illustrated lessons delivered in the classroom: 1) Introduction to environment, 2) Biodiversity and habitat, 3) Indonesian, Sulawesi, and Tangkoko flora and fauna, 4) Primates, crested macaques and the Macaca Nigra project, 5) Trip to Tangkoko forest, 6) Conservation issues and solutions, and 7) Marine biodiversity. These lessons are coupled with games, hands-on activities, quizzes, as well as a field trip to the Tangkoko forest. An English version of our fourth lesson is available online: http://online.fliphtml5.com/tngqz/ahjj/#p=1.

We evaluated 10 years of quantitative data (2011–2021) for the conservation education school programme run by TCE. We provided questionnaires to pupils 1 month before and 1 month after our school interventions to assess whether our conservation education programme has an impact on their knowledge, habits, and behaviour towards their local environment. We compared the overall scores of participants before and after taking part in the programme, as well as their scores in the three sections of the questionnaire quantifying knowledge, habits, and behaviour. If our programme had a positive impact on the participants’ knowledge, habits, and behaviour after taking part in our interventions, we predict that we would find higher scores in each of these three categories when assessed 1 month after the school interventions compared with 1 month before. In addition, we investigated whether the success of our programme depends on age, gender, residence, and parents’ occupation. Finally, we explored whether participating multiple times increased individuals scores. If repetition facilitates information retention and promotes proconservation attitudes, we predict that pupils who took part in our education programme several times will show higher scores than pupils who have participated only once.

## Methods

### Data Collection

We evaluated the questionnaires provided to the participants of our school programme 1 month before and 1 month after they received the seven lessons delivered by TCE’s local Indonesian staff between 2011 and 2021 (except 2019–2020 year, when interventions were cancelled due to the COVID 19 pandemic). In total, we collected data from 1,183 pupils from 106 classes and 28 schools from 60 villages. These pupils participated in the TCE programme when they were in primary school (Year 4, 5, and 6, mean ± SD age = 10.3 ± 1.1 y) junior secondary school (Year 7 and 8, mean ± SD age = 12.7 ± 0.9 y), or senior secondary school (Year 10 and 12, mean ± SD age = 15.3 ± 0.7 y). Most pupils participated in the programme once (*N* = 1,155), but 27 pupils participated twice, and one pupil participated three times.

This evaluation is quantitative in nature and is implemented through paper-written questionnaires in the pupils’ native language (Bahasa Indonesia), which we deliver to participants in schools, assessing their knowledge, e.g., “Write the name of the animal (under pictures of 6 animals living in Sulawesi and other islands), and tick the appropriate box if you think this animal lives in Sulawesi or not”; “Write the names of 3 animal species who live in your surroundings (Tangkoko, Duasudara, Tumpa, Klabat).” To evaluate habits, we asked questions, such as “Does your family have any wildlife as a pet?”; “Does your family eat wildlife?” To assess pupils’ behaviours, we asked questions, such as “When you are in the forest, do you approach macaques?”; “When you are in the forest, do you feed macaques?” (See electronic supplementary materials for the full questionnaire.) The local Indonesian TCE team and the teachers provided the questionnaires in class. Pupils mostly answered the questions independently. Sometimes, the TCE team and the teachers read the questions to the pupils, answered possible questions that the pupils had, or wrote down the answers when needed, without influencing the answers. There was no time limit to complete the questionnaires, but most were completed within an hour.

Although some questions were worded slightly differently across years, we focused on questions that were in all the questionnaires throughout this study period. The questionnaires included the following sections: Demographic information (e.g., gender of participant, parents’ job, residence of participants’ family, age), knowledge of local biodiversity (20 closed and open-ended questions, with a maximum section score of 23 points), habits involving local biodiversity (4 closed questions with a maximum section score of 4 points), and behaviour when encountering crested macaques in the forest (4 closed questions with a maximum section score of 7 points) (see supplementary materials for details).

In addition to the basic demographic information, we created a variable combining pupils’ level, their school, and the year they participated in the programme. Pupils lived in various locations across the North Sulawesi area, especially around the main cities of Bitung, Airmadidi, and Manado. We categorised the residence of participants’ family into rural, suburban, and urban. We designed these categories according to the following criteria defined by the Indonesian Statistics Office (Badan Pusat Statistik): topography, population density per km^2^, percentage of agricultural households, and presence of or access to urban facilities. If the locations fell under a score of 10, we defined them as rural; if the score was ≥ 10, we defined them as urban (Indonesian Statistics Office, 2010). We categorised the occupations of the participants’ fathers into the following categories: managers and professionals, semiprofessionals, routine nonmanual workers, and manual workers. Because most of the mothers were not in employment (83%), we did not categorise mothers’ occupations. Lastly, the questionnaires included a time at intervention variable (hereafter “time”), indicating whether the questionnaire was taken before or after the pupil had participated in the TCE programme.

After collecting the questionnaires, the local team scored each question for each participant. They scored some questions (typically closed questions that could be answered by “Yes” or “No”) as either 0 (wrong answer) or 1 (right answer), and others (e.g., questions from the habits section that could be answered by “Never,” “Sometimes,” “Every time”) as 0 (wrong answer), 1 (approximate answer), or 2 points (right answer). For the knowledge section, we assumed that when a question was left unanswered, it was most likely because the pupil did not know the correct answer and scored missing answers as incorrect (0 point). Higher scores reflected better knowledge about the local environment (e.g., recognising and accurately naming emblematic Indonesian and Sulawesi animals based on pictures), higher proenvironmental habits (e.g., answering that they have not consumed wildlife), and better behaviour (e.g., answering that it is wrong to approach macaques in the forest).

### Data Analysis

We scaled the scores by dividing them by the maximum possible number of points for the corresponding section (knowledge: 23, habits: 4, behaviour: 7) to have all section scores between 0 and 1. Then, we calculated a total score for each questionnaire, corresponding to the mean of the scores of the three sections (knowledge, habits, and behaviour).

There were 6% missing values in the habits section and 24% in the behaviour section. The knowledge section did not contain missing values, because we scored missing answers as incorrect. For the demographic information, if a value was missing in only one of the two questionnaires taken by a pupil (before and after the programme), we retrieved it from the other one, assuming that the values were the same. This meant that we had no missing values for gender, class, or school. However, 26% of the values for fathers’ job categories, 8% for residence and 3% for age were still missing. In total, 1,432 of 2,384 questionnaires (60%) were incomplete. In most cases, we think values were missing, because pupils forgot to answer a question or wrote an unintelligible answer, which was ignored during the digitization of the data. We also accidentally lost most of the data for fathers’ job categories for the year 2017–2018 before digitalization. We have no reason to believe that pupils intentionally left questions unanswered. We thus considered the missing data to be missing at random. To our knowledge and based on inspection of the means and the proportions of the demographic information, there were no obvious differences between pupils with complete and incomplete questionnaires.

### Statistical Analyses

Given that we had a considerable proportion of incomplete data, we used multiple imputation to fill the missing data with substituted values. This method is well established in psychological and clinical research to handle item-level missing data in questionnaires (Enders, 2017). It allows the use of incomplete datasets without throwing away information and is considered to be the best method to deal with missing data (van Ginkel *et al*., 2020). If used correctly, another advantage is that it avoids biases associated with alternative methods like listwise-deletion or simple imputation, such as biased *p*-values or confidence intervals. The main drawback of using multiple imputation is that it takes time to run the analysis, and it requires further efforts and thinking in addition to the statistical analyses per se. Multiple imputation consists of three main steps (van Ginkel *et al*., 2020). First, for each missing data point, a statistical model uses information from other variables in the dataset to generate plausible values, while integrating a random error component. This results in the creation of different complete imputed datasets, each containing slightly different values for the imputed data, thus reflecting the uncertainty around their true values. Then, standard statistical analyses are performed on each of the imputed datasets, resulting in slightly different outcomes. Lastly, the parameter estimates of each analysis are combined into the overall result.

We imputed all the variables for which we had missing values, i.e., age, residence of participants’ family, father’s job category, and the individual scores for the questions in the habits and behaviour sections by using item-level imputation to generate 20 multiple imputed datasets. We conducted multiple imputation in R version 4.0.3 (R Core Team, 2021) by using the package “MICE” version 3.14.0 (van Buuren & Groothuis-Oudshoorn, 2011). To impute the missing data, we included “gender,” “age,” “village category,” and “father’s jobs category” as predictors for all the incomplete variables. We used the “quickpred” function, which automatically selects additional predictors for each incomplete variable. The number of predictors per incomplete variable ranged from 5 to 23, with a mean of 13.6 predictors. We used an appropriate imputation method for each type of incomplete variable: predictive mean matching for numerical variables, logistic regression for binary variables, proportional odds model for ordinal variables, and polytomous logistic regression for nominal variables (van Buuren, 2018). After conducting the multiple imputation, we checked for convergence of the algorithm by plotting the mean and the standard deviation of the synthetic values of the imputed variables against the iteration number. With the number of iterations per imputation set at 15, the MICE algorithm seemed to converge satisfyingly. We also checked for discrepancies between observed and imputed data by visual inspection of side-by-side, box-and-whisker plots and of kernel density estimates of the imputed and observed data. We detected no discrepancies between observed and imputed data. For each of the imputed datasets, we then calculated the score for the habit and behaviour sections and the total score for the questionnaires containing missing variables in these sections prior to multiple imputation.

We built all the statistical models in R version 4.0.3 (R Core Team, 2021). To assess the impact of the TCE programme on the scores of the different sections of the questionnaire (knowledge, habit, behaviour), we fitted three Cumulative Link Mixed Models (CLMM), using the function “clmm” of the package “ordinal” version 2019.12–10 (Christensen, 2022). In each CLMM, we used the score of one of the sections as the response variable. To evaluate the effect of the programme on the total score of the students that participated in the program once, we fitted one Linear Mixed Model (LMM), using the function “lmer” of the package “lme4” version 1.1–26 (Bates *et al*., 2015), with the total score as the response variable. For each model (CLMMs and LMM), we included time (before or after the programme), gender (boy, girl), and age as fixed effects test predictors. As control variables, we added the father's job and residence categories. For both the CLMMs and the LMM, we z-transformed age to a mean of zero and a standard deviation of one for each of the imputed datasets before fitting the models. We included Pupil’s ID and Class as random effects control predictors in all models. We executed all the analyses for each of the 20 imputed datasets and then estimated the parameters of substantive interest in each imputed dataset separately. Lastly, we combined these parameters using Rubin’s rules (Rubin, 2004), which is the most used method to pool results from analyses of multiply imputed data, with the function “pool” of the “MICE” package version 3.14.0 (van Buuren & Groothuis-Oudshoorn, 2011).

For all the models, we tested the overall effect of all test predictors on the response variable using a Likelihood Ratio Test (LRT) by comparing the full model with a null model comprising only the random effects. We used the “anova” function of the “MICE” package, with the method argument set to “D2” and the “use” argument set to “likelihood” to pool the LRT statistics from the repeated analyses.

For comparison, we also performed the analyses on the subset of complete cases (i.e., questionnaires with no missing values). To the best of our knowledge, there is no built-in function to check for collinearity in multiple imputed repeated analyses. Therefore, we determined Variance Inflation Factors (VIF) for the complete dataset by creating corresponding linear mixed models using the same test predictors, control variables and random effects as in our CLMMs using the function “vif” of the package “car” (version 3.0) (Fox *et al*., 2022). This revealed collinearity to be no issue, as VIFs were around 1 for all the models. We determined model stability for the complete cases analyses by dropping raters one at a time, fitting the full model to each of the subsets obtained, and then comparing the estimates obtained for the subsets with those obtained for the full data set, using two self-written R functions provided by Roger Mundry (biostatistician at the German Primate Center). This showed that the model was stable.

For these analyses, we only included pupils who participated once in the programme. We performed descriptive analyses for the few students who took part in TCE’s interventions on several occasions because the sample size was too small for formal analysis.

## Ethical Note

The research was approved by the Department of Psychology ethics committee at the University of Portsmouth. The research also adhered to the legal requirements of Indonesia. The Department of Education of the Municipality of Bitung as well as the Nature Conservation Agency of North Sulawesi (BKSDA Sulawesi Utara) gave permission to conduct the Tangkoko Conservation Education programme.

## Results

### Descriptive Statistics

The mean age of the pupils that participated in the TCE programme was 11 years (SD = 1.66): 36% of the participants were boys, and 64% were girls; 5% resided in urban areas, 27% in suburban areas, and 68% in rural areas; 3% of the pupils’ fathers were managers and professionals, 11% were semiprofessionals, 17% were routine non-manual workers, and 69% were manual workers.

### Pupils who Took Part in TCE’s Interventions Only Once

With our full-null model comparisons for pupil taken part once testing the combined impact of time, gender, age, father’s job category and location, we found a clear impact of the test predictors on the total score (LMM LRT: χ^2^ = 7.19, df = 8, *p* < 0.001), the score for behaviour (CLMM: LRT for behaviour score: χ^2^ = 2.89, df = 8, *p* < 0.01) and the score for knowledge (CLMM: LRT for knowledge score: χ^2^ = 9.19, df = 8, *p* < 0.001), but not on the score for habits (CLMM: LRT for habit score: χ^2^ = 1.29, df = 8, *p* = 0.244). Therefore, we did not consider predictors of the score for habits further.

**Fig. 1:**
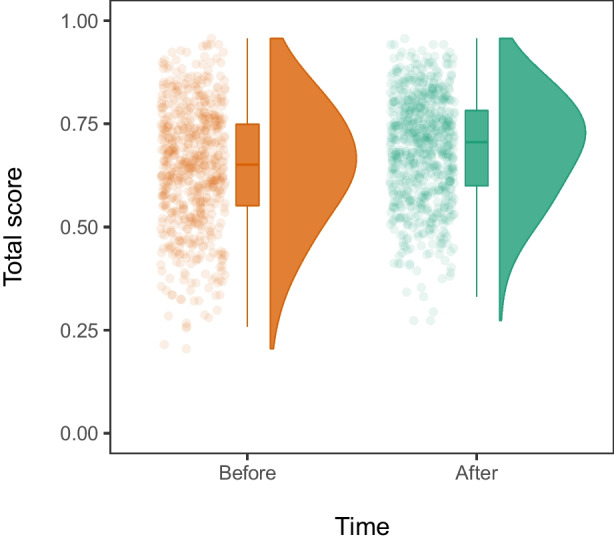
Pupils’ total scores before (orange) and after participation (green) in Tangkoko Conservation Education interventions between 2011 and 2021 in the Greater Tangkoko area, North Sulawesi, Indonesia. Dots show the individuals’ scores. Boxplots show the first and third quartile of observed values and the line shows the median. Whiskers extend to 1.5 times the interquartile range above the third and below the first quartile. The distribution on the right of the boxplots shows the distribution of participants’ scores.

Time had a significant effect on the total, behaviour, and knowledge scores, with the pupils scoring higher after their participation in the TCE programme compared with before (total scores: Table I and Fig. 1a, scores on behaviour: Table II and Fig. 1b and c, scores on knowledge: Table III and Fig. 1d).

**Table I:** Results of a linear mixed model testing the influence of pupils’ age (z-transformed), gender (boy, girl), time (before or after Tangkoko Conservation Education interventions), village category (rural, suburban, or urban) and father’ jobs category (manual worker, professional, routine nonmanual worker, or semiprofessional) on their total score for the evaluation questionnaire (mean of the scores for the knowledge, habit, and behaviour sections) between 2011 and 2021 in the Greater Tangkoko Area, North Sulawesi, Indonesia. *P*-values < 0.05 are in bold

Term	Estimate	S.E	χ^2^	df	*p*
Age	0.0043	0.0049	0.87	1095.81	0.38
Gender	−0.012	0.0072	−1.64	2028.72	0.10
Time	0.026	0.0037	7.20	905.99	**<0.001**
Residence: suburban^a^	−0.0093	0.014	−0.69	336.72	0.49
Residence: urban^a^	−0.015	0.019	−0.76	209.42	0.45
Father’s job category: professional^b^	0.0024	0.019	0.13	239.56	0.90
Father’s job category: routine, nonmanual worker^b^	−0.00093	0.011	−0.088	154.45	0.93
Father’s job category: semiprofessional^b^	0.013	0.013	1.031	294.63	0.30

^a^Reference level is rural

^b^Reference level is manual worker

**Table II:** Results of a Cumulative Linear Mixed Model testing the influence of pupils’ age (z-transformed), gender (boy, girl), time (before or after Tangkoko Conservation Education interventions), village category (rural, suburban, or urban), and father’ jobs category (manual worker, professional, routine nonmanual worker, or semiprofessional) on their knowledge score between 2011 and 2021 in the Greater Tangkoko Area, North Sulawesi, Indonesia. *P*-values < 0.05 are in bold

Term	Estimate	S.E	χ^2^	df	*p*
Age	0.061	0.078	0.78	1724.25	0.44
Gender	0.10	0.10	0.98	2332.79	0.33
Time	0.42	0.052	8.015	2344.90	**<0.001**
Residence: suburban^a^	−0.18	0.24	–0.76	225.69	0.45
Residence: urban^a^	−0.18	0.30	–0.60	191.44	0.55
Father’s job category: professional^b^	0.14	0.27	0.51	492.05	0.61
Father’s job category: routine, nonmanual worker^b^	−0.22	0.15	-1.41	166.15	0.16
Father’s job category: semiprofessional^b^	0.12	0.18	0.64	400.72	0.52

^a^Reference level is rural

^b^ Reference level is manual worker

**Table III:** Results of a Cumulative Linear Mixed Model testing the influence of pupils’ age (z-transformed), gender (boy, girl), time (before or after Tangkoko Conservation Education interventions), village category (rural, suburban, or urban) and father’ jobs category (manual worker, professional, routine non-manual worker, or semiprofessional) on their behaviour score between 2011 and 2021 in the Greater Tangkoko Area, North Sulawesi, Indonesia. *P*-values < 0.05 are highlighted in bold

Term	Estimate	S.E	χ^2^	df	*p*
Age	−0.018	0.060	−0.29	809.44	0.77
Gender	−0.26	0.10	−2.59	817.81	**<0.001**
Time	0.21	0.060	3.48	464.59	**<0.001**
Residence: suburban^a^	−0.063	0.16	−0.40	324.73	0.69
Residence: urban^a^	−0.37	0.27	−1.37	136.26	0.17
Father’s job category: professional^b^	−0.050	0.29	−0.18	156.50	0.86
Father’s job category: routine, nonmanual worker^b^	0.11	0.16	0.71	95.41	0.48
Father’s job category: semiprofessional^b^	0.10	0.19	0.53	157.49	0.60

^a^Reference level is rural

^b^Reference level is manual worker

**Fig. 2:**
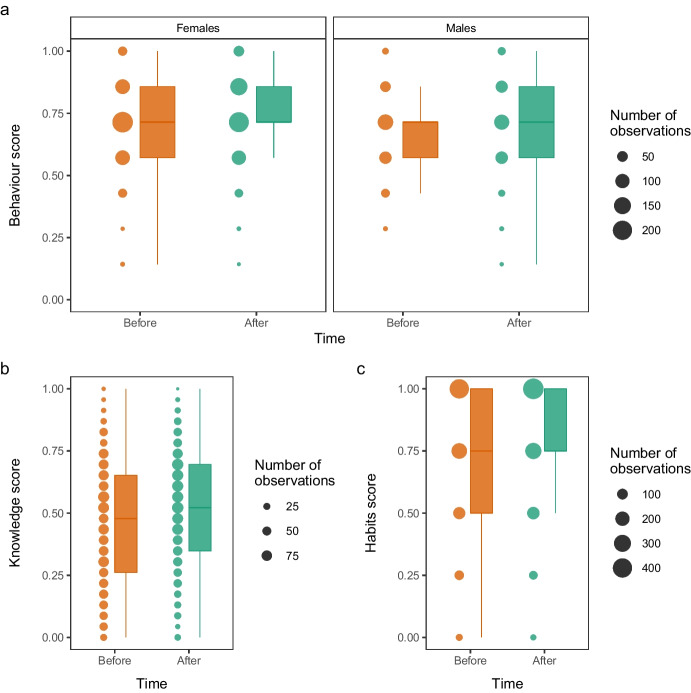
Pupils’ scores in **a**) behaviour, **b**) knowledge, and **c**) habits subscales before (orange) and after participation (green) in Tangkoko Conservation Education interventions between 2011 and 2021 in the Greater Tangkoko Area, North Sulawesi, Indonesia. Dots show the participants’ scores, with the size of the circle referring to the number of observations. Boxplots represent the first and third quartile of observed values and the line shows the median. Whiskers extend to 1.5 times the interquartile range above the third and below the first quartile.

Gender affected the score for behaviour significantly; girls scored higher than boys for this section (Table III and Fig. 2a). Gender did not influence total score (Table I) or the score for the knowledge section (Table II). Neither age, location nor father’s job category had a significant effect on any of the calculated scores.

We obtained similar results when fitting the models with the data from complete questionnaires only, without using multiple imputations (see ESM for details).

### Descriptive Analyses for Students Who Took Part in TCE’s Interventions Several Times

**Fig. 3:**
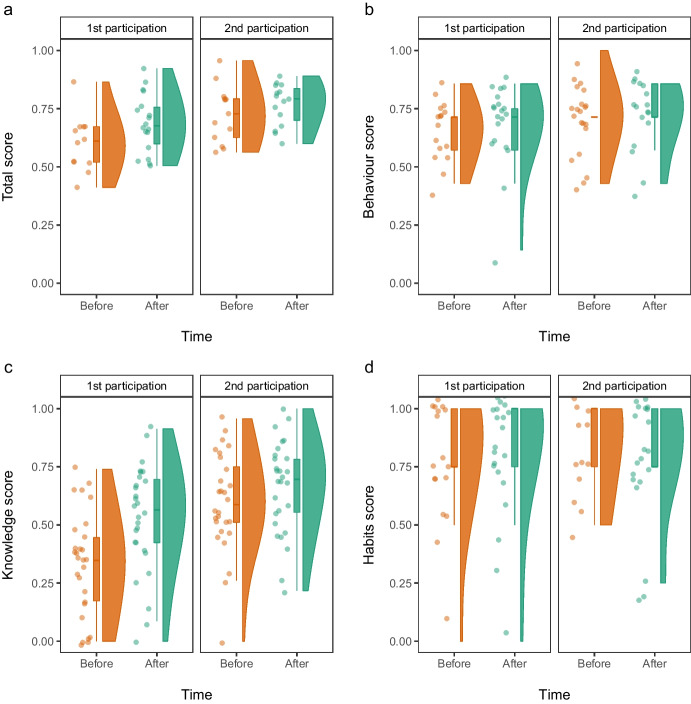
Pupils’ **a**) total scores, scores in **b**) behaviour, **c**) knowledge, and **d**) habits subscales before (orange) and after participation (green) in their first and second participation in Tangkoko Conservation Education interventions between 2011 and 2021 in the Greater Tangkoko Area, North Sulawesi, Indonesia. Dots show the participants’ scores, with the size of the circle referring to the number of observations. Boxplots represent the first and third quartile of observed values and the line shows the median. Whiskers extend to 1.5 times the interquartile range above the third and below the first quartile.

Very few pupils (*n* = 28) took part in the programme and answered the questionnaire more than once (twice: *N* = 27, thrice: *N* = 1). Visual inspection of the scores of the questionnaires taken in participants first and second participation in the programme suggests that pupils had a higher score before their second participation than before their first participation (Fig. 3). Pupils’ total score and knowledge scores were marginally higher after compared with before their second participation (Fig. 3a and c).

## Discussion

Our most important result is the significant increase in children’s knowledge and behaviour scores after their participation in the programme. Their habits scores also increased, but this increase was not statistically significant. When considering gender, girls scored significantly higher than boys in terms of behaviour towards wildlife. Other factors, such as age, father’s job, and residence of participants’ family, did not affect pupils’ scores significantly. Children who participated in the programme more than once seemed to obtain higher scores in their second participation, although the sample size was too small for formal analysis.

As we predicted, pupils’ scores increased significantly after the TCE interventions compared with their score before the interventions. This was true for knowledge and behaviours but not habits. These results corroborate similar studies showing an increase in scores regarding either knowledge, behaviours, or attitudes when pupils participate in primate conservation education programmes. For example, a similar primate education programme based in Colombia reported an increase in children’s knowledge and awareness about cotton-top tamarins (*Saguinus oedipus*) after their participation in education interventions (Savage *et al*., 2010). In Uganda, participation in a conservation education programme resulted in an increase in children’s scores for questions assessing knowledge, positive attitudes, and behaviours around the Kalinzu Forest Reserve (Kuhar *et al*., 2010). A meta-analysis summarised five decades of environmental education programmes worldwide and emphasised the importance of focussing on behaviour change in response to the current crises of climate change and species extinction for future programmes (van de Wetering *et al*., 2022). The TCE programme aligns with this recommendation and seems to have a clear positive impact on participants’ behaviour, which is encouraging for the future of the local endangered species. Still, assessments of the impact of the programme over the longer-term (e.g., 6 months or 1 year after completing the programme), coupled with quantitative and qualitative data on the participants’ engagement with proenvironmental behaviour are required to draw firm conclusions regarding the efficacy of TCE’s actions in this respect.

In our study, participants' habits scores were higher after compared with before the programme, but this increase was not statistically significant. The range of possible scores (from 0 to 4) was lower than for knowledge (0–23) and behaviour (0–7), and participants often reached the maximum score before the interventions, possibly masking any significant increase over time via a ceiling effect. It also is possible that our programme failed to change the children’s habits. Habits are notoriously difficult to assess (Linder *et al*., 2022) and can take longer to change than knowledge, as they are linked to cultures, tradition, and economic constraints. A study suggests that a change of habits requires participants to spend a significant amount of time thoroughly studying local conservation issues to allow greater reflection and engagement (Stevenson, 1997). This method can help students to have a sense of ownership regarding an issue and make recommendations to solve it. We need to improve this aspect of our teaching methods and questionnaires: first by spending more time focusing on and discussing local conservation issues with the students, and by adding more questions related to habits to assess this component more efficiently in the future.

We predicted that girls would obtain better results than boys, and our study showed that this was the case regarding their behaviour. This result is in line with those of several studies demonstrating that women show higher levels of positive behaviours and attitudes than men when interacting with wildlife (Herzog, 2007; Zelezny *et al*., 2000). For example, according to a review on gender differences in human–animal interactions, women seem to be more aware and sensitive to animal welfare and protection, whereas men seem to engage in more negative behaviours towards wildlife, such as hunting (Herzog, 2007). On a larger scale, this review shows that women are significantly more active in grassroots environmental campaigns and programmes than are men. Although we lack systematic data about hunting or logging in the area, we can relate to our own experience when encountering hunters in the Tangkoko forest, MNP staff mostly report that men (children or adults) operate such activities and not women (MNP, personal observations). Nevertheless, despite scoring higher than boys in the behaviour part of our questionnaire, girls might attach just as much importance to hunting as men do. In North Sulawesi, where traditionally, wildlife is hunted for consumption in important celebrations, and therefore, has strong cultural value, this might indeed be the case. Additional research is needed to fully understand the influence of gender on behaviour scores.

Older pupils did not perform better than younger ones. It seems surprising that there is no difference at least in knowledge, because pupils in Indonesia have a biology curriculum related to some of the concepts taught alongside our environmental lessons. It is possible that the content of TCE lectures can be understood by young and old pupils equally well, so this may inhibit an age effect. Therefore, we need to better adapt our environmental lessons according to the age of participants (whether they are in primary or secondary schools). Our environmental lessons are not included in the regional curriculum yet, although we are currently working on a collaboration with the Department of Education of North Sulawesi to achieve this. Currently, only a couple of local teachers implement our lessons as part of their biology curriculum. A conservation education programme called YANI (https://nantuforest.org), based in Gorontalo Province near North Sulawesi, has been successful in developing Conservation Curriculum Materials on a provincial level for primary school children. Thus, it is important for the TCE programme to continue to lobby the local and regional government to include our environmental lessons in the pupils’ biology curriculum on a provincial level so pupils can know more about their local wildlife and environment as they grow up and reach a higher education level. In the meantime, we run a Science Camp programme targeting senior secondary school students, with the goal of deepening their knowledge and awareness about primate conservation, research, and education.

Our study has several limitations. Because of limited time, financial and human resources, it does not involve control groups of pupils who do not participate in the TCE programme. Therefore, we cannot rule out the possibility that the pupils’ scores increased due to external factors, such as involvement in other conservation education programmes or maturation. Pilot data available on small sample sizes in 2013 and 2018 yielded contradictory results (Tangkoko Conservation Education, unpublished data). In 2013 (*N* = 12), knowledge scores increased significantly in the control as well as the experimental group, but this increase was smaller in the control group. In 2018 (*N* = 23), none of the scores increased in the control group. With such small sample size, however, it is difficult to draw firm conclusions. To corroborate our results, further research is needed using both intervention pupils and an equivalent control group of pupils who are not part of the TCE programme.

Similarly, our evaluation is limited to specific time points, within a month of the conservation education programme. Behavioural change takes time and is reinforced by repeated engagement in various activities. On one occasion where resources were available, we tested the same participants 6 months and 1 year after they had completed the programme (Chanvin *et al*., unpublished MRes dissertation). Using similar questions, pupils’ knowledge scores increased immediately after participation and remained stable after 1 year. Although only a few participants were included in this study (*N* = 16), it shows that the programme can have a positive impact on children many months after their participation. While promising, these results need to be confirmed by a larger scale study, specifically designed to test the long-term effect of our programme.

The use of questionnaires only to assess behaviour change is also potentially problematic. We cannot rule out the possibility that pupils answer in a way that is expected from them or that appear more desirable for the people conducting the evaluation (i.e., demand characteristics: Orne, 1996). A possible way of mitigating this potential issue would be to combine the questionnaire evaluations with in-depth interviews and observations of the participants. This combined approach should allow researchers to determine whether participants’ answers to the questionnaire correspond to actual behaviour change. However, this would require a lot of time and resources which are not currently available to TCE and rarely available to conservation education organisations in general.

Although we had a section related to pupils’ attitude measurement in our questionnaires, we did not take it into account in this study because we had only two statements related to this topic where children answered through a scale ranging from “strongly disagree” to “strongly agree.” The statements were quite biased towards a positive answer (“I believe that I have the capacity to protect wildlife”) and pupils often answered positively to these questions before and after the interventions, making it difficult to assess this component objectively. One recommendation is to add more and better adapted attitudes questions in future surveys to evaluate this important aspect of conservation education.

Despite these limitations, we think that the TCE programme is successful in changing various aspects of participants’ perceptions regarding their local environment. Their knowledge increased, and they are willing to change their behaviour once they have participated in the TCE programme. Therefore, we conclude that we need to continue to deliver our TCE activities—the only regular education programme aimed at local school children since 2011. These results are important to assess what aspects of the programme and its evaluation need to be improved to deliver more efficient interventions. We need to systematically include a control group of students each year of implementation to test whether TCE lessons have a positive impact on the students who participate in it compared with students who do not participate. We need to review our lesson content to improve the pupils’ habits scores after their participation in the programme. For example, we need to focus more on the content of our 6th lesson (“Conservation issues and solutions”) and make it more interactive and thoughtful for students to have in-depth knowledge and reflection about topics that are local and relevant to them. This lesson also can help to focus on male participants, because they seem to be more involved in hunting/setting traps in the Tangkoko forest than female participants (MNP, personal observations). Discussing such sensitive topics with them in an interactive and open-minded way could help them to find their own alternative solutions to use natural resources (rather than hunting, setting traps, or logging). As a result of these changes and adaptations to how we teach our lessons and their content, we can be hopeful that students’ habits can significantly change over time, thus gradually reducing the pressure on local wildlife. We need to pay specific attention to older students so the content of our lessons can be adapted to their curriculum and can increase their knowledge compared with younger students. To help with this matter and receive the full support of local schools to deliver our lessons in a formal setting, it is important to include our lessons in the regional curriculum of North Sulawesi, which we aim to do within the next 3 years. Future studies should relate the outcome of the evaluation to tangible measures of conservation success, such as the number of traps found in the forest and population trends of crested macaques in the areas where TCE operates.

## Conclusions

Our results showed that pupils’ knowledge and behaviour scores increased significantly after participating in our conservation education programme at least once. Habits score also increased, but this increase was not statistically significant. Girls scored significantly higher than boys in terms of positive behaviour towards wildlife, whereas age and socioeconomic background (fathers’ profession and residence category) showed no significant effect. Overall, we provide evidence of a positive effect of our programme and identify areas of improvement. Our findings support the recommendation to systematically evaluate the effectiveness of conservation education programmes (Jacobson, 2010; Jacobson & McDuff, 1997), with the goal of informing and engaging the local community in the protection of endangered species.

## Supplementary Information

Below is the link to the electronic supplementary material.

Supplementary file1 (DOCX 125 KB)

## Data Availability

The data are available on figshare: 10.6084/m9.figshare.22147517.
