# Supplementary material for: Ten Years of Positive Impact of a Conservation Education Program on Children's Knowledge and Behaviour Toward Crested Macaques (Macaca nigra) in the Greater Tangkoko Area, North Sulawesi, Indonesia
Source: Int J Primatol. 2023 May 3:1–21. Online ahead of print. doi: 10.1007/s10764-023-00356-9 (PMC10155166; doi:10.1007/s10764-023-00356-9)
Supplement: Supplementary file 1 — Supplementary file1 (DOCX 125 KB) [file 10764_2023_356_MOESM1_ESM.docx]

**Supplementary materials:**

**Ten years of positive impact of a conservation education program on children's knowledge and behaviour towards crested macaques (*Macaca nigra*) in the Greater Tangkoko Area, North Sulawesi, Indonesia.**

Mathilde Chanvin, François Lamarque, Nona Diko, Muhammad Agil, Jérôme Micheletta, Anja Widdig

**Questionnaire:**

| **Pupil N°: ____** |
| --- |

**TANGKOKO CONSERVATION EDUCATION** INTERVENTION GROUP

| Name |  | Class |  | |
| --- | --- | --- | --- | --- |
| School |  | Area |  | |
| Gender |  | Age |  | |
| Father’s job |  | Mother’s job |  |  |

Have you already participated in our programme (Tangkoko Conservation Education?) 🞏 Yes 🞏 No

Have you already participated in another conservation education programme? 🞏 Yes 🞏 No

**HABITS:**

You go to the forest: 🞏 Never 🞏 Once a year 🞏 Every month🞏 Every week 🞏 Everyday

🞏 Alone 🞏 With your friends 🞏 With your family

What do you do when going to the forest?..................................................................................................

Does your family take some wood from the forest? 🞏 Yes 🞏 No

If yes, what do you use it for? ….........................................................................................

Does your family eat fruits from the forest? 🞏 Yes 🞏 No

Does your family eat wildlife? 🞏 Yes 🞏 No

If yes, which animal? 🞏 Rat 🞏Wild pig 🞏 Cuscus 🞏 Snake 🞏 Bat 🞏 Macaque 🞏Bird Other:.......................................

Does your family use forest products for traditional medicine? 🞏 Yes 🞏 No

Does your family have any wildlife as pet? 🞏 Yes 🞏 No

If yes, which one? 🞏 Cuscus 🞏 Macaque 🞏 Deer 🞏 Wild pig 🞏Bird Other:.......................................

If you don’t have any wildlife as pet, would you and your family wish to have one? 🞏 Yes 🞏 No

If yes, which one? 🞏 Cuscus 🞏 Macaque 🞏 Deer 🞏 Wild pig 🞏Bird Other:.......................................

**BEHAVIOUR:**

**WHEN YOU ARE IN THE FOREST:**

In the forest, do you throw your rubbish: 🞏 Everywhere 🞏 In the bin

Have you already seen a crested macaque in the forest? 🞏 Yes 🞏 No

**ABOUT CRESTED MACAQUE:**

Do you see macaques when you are in the forest? 🞏 Each time 🞏 Sometimes 🞏 Never
If yes, are you happy? 🞏 Yes 🞏 No 🞏 I don’t care

Do you approach them? 🞏 Each time 🞏 Sometimes 🞏 Never
Do you observe them? 🞏 Each time 🞏 Sometimes 🞏 Never
Do you feed them? 🞏 Each time 🞏 Sometimes 🞏 Never
Are you afraid of macaques? 🞏 Each time 🞏 Sometimes 🞏 Never

If you don’t like to meet macaques, what do you do?

Chase them 🞏 Keep distance 🞏 Run from them🞏

Other please explain:

**IN THE GARDEN:**

Do you have a garden? 🞏 Yes 🞏 No

If yes, do macaques come to your garden? 🞏 Each time 🞏 Sometimes 🞏 Never
Are you happy to see macaques next to your garden? 🞏 Yes 🞏 No

If yes, do you approach them? 🞏 Each time 🞏 Sometimes 🞏 Never
Do you observe them? 🞏 Each time 🞏 Sometimes 🞏 Never
Do you feed them? 🞏 Each time 🞏 Sometimes 🞏 Never

**IN THE VILLAGE:**

Do you care about the rubbish? 🞏 Yes 🞏 No

Do you see wildlife in your house/yard? 🞏 Yes 🞏 No

If yes, which ones? 🞏 Rat 🞏Wild pig 🞏 Cuscus 🞏 Snake 🞏 Bat 🞏 Macaque 🞏Bird Other: ……………………………

What do you do with them: Chase them 🞏 Catch them 🞏 Hunt them 🞏 Nothing 🞏Other: ……………………………

**KNOWLEDGE ABOUT LOCAL ENVIRONMENT:**

There are three important elements for the development of life. Water is one of them. Write the two other elements: ……………………and ………………………………..


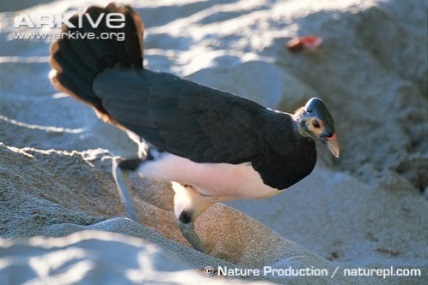

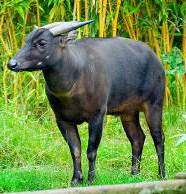

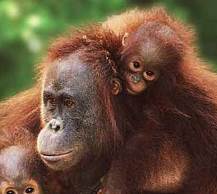

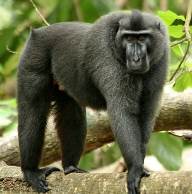

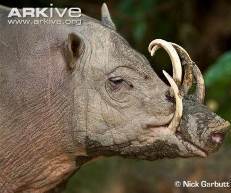

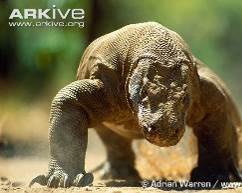
Write the name of the animal and tick the approriate box if you think this animal lives in Sulawesi or not:
……………………………… …..……………………… ………………………. ……………………………. .…………………….

………………….

Lives in Sulawesi? Lives in Sulawesi? Lives in Sulawesi? Lives in Sulawesi? Lives in Sulawesi? Lives in Sulawesi? □ Yes □ No □ Yes □ No □ Yes □ No □ Yes □ No □ Yes □ No □ Yes □ No

Choose:
Tangkoko-Duasudara is a : 🞏 Nature Reserve 🞏Protected Forest 🞏National Park 🞏Recreational park

Batuangus is a : 🞏 Nature Reserve 🞏Protected Forest 🞏National Park 🞏Recreational park

Gunung Klabat is a  : 🞏 Nature Reserve 🞏Protected Forest 🞏National Park 🞏Recreational park

Gunung Tumpa is a  : 🞏 Nature Reserve 🞏Protected Forest 🞏National Park 🞏Recreational park

Bunaken island is a: 🞏 Nature Reserve 🞏Protected Forest 🞏National Park 🞏Recreational park

According to you, the crested macaque is:

Protected by law: 🞏 Yes 🞏 No
Critcally endangered: 🞏 Yes 🞏 No

Very important for the forest regeneration 🞏 Yes 🞏No

Which animal group contains the largest amount of species?

🗖Mammals 🗖Insects 🗖Birds 🗖Amphibians 🗖Reptiles

Write 3 activities to protect forest and wildlife:

- ……………………………………………………………………………………………….
- ……………………………………………………………………………………………….
- ……………………………………………………………………………………………….

Why is Sulawesi’s biodiversity special compared to other Indonesian islands? Choose two:
🗖Because there are many endemic wildlife 🗖Because there are Orangoutans and tigers
🗖Because there are a lot of Nature Reserves 🗖Because there are 7 macaque species

Write the names of 3 animal species who live in your surroundings (Tangkoko, Duasudara, Tumpa, Klabat)
……………………………………………………………………………………………………………………………………………………………………………………

**ATTITUDES:**

Are you interested in protecting the nature around you? (such as wildlife, plants, trees..) 🗖Happy 🗖So-so 🗖Not happy

If you are not happy, explain why: ………………………………………………………………………………………………………………………………

Are you interested in being part of Tangkoko Conservation Education programme?
If you are not happy, explain why: ………………………………………………………………………………………………………………………………

What do you think? (tick the box)

- Your capacity is determined by how smart you are
  🗖Agree 🗖Somewhat agree 🗖Neither agree or disagree 🗖Somewhat disagree 🗖Disagree
- You can learn new things, but you can’t change your basic intelligence:
  🗖Agree 🗖Somewhat agree 🗖Neither agree or disagree 🗖Somewhat disagree 🗖Disagree
- I believe I have the capacity to protect the environment
  🗖Agree 🗖Somewhat agree 🗖Neither agree or disagree 🗖Somewhat disagree 🗖Disagree

**Additional analysis on complete questionnaires (N=952):**

We obtained similar results when fitting the models with the complete, non-imputed datasets. Similar to the models fitted the imputed datasets, we found a clear impact of the test predictors on the total score, the score for behaviour and the score for for knowledge, respectively (LMM LRT for total score: χ2 =53.32, df = 8, p < 0.001; CLMMs: LRT for behaviour score: χ2 =16.42, df = 8, p < 0.05; LRT for knowledge score: χ2 =76.85 , df = 8, p < 0.001). Again, the full-null model comparison for the scores for habits revealed that the test predictors did not have a significant effect on the score for habits (CLMM: LRT for habit score:  χ2 = 6.95, df = 8, p > 0.05).  The models fitted with the complete datasets showed that all the scores were significantly higher after participation in the TCE (see Tables 1, and 3). It also showed that female pupils scored higher than male pupils for the behaviour section (see Table 3). The only difference with the models fitted with the multiply imputed datasets was that pupils with fathers working as routine non-manual worker had a lower score for the knowledge section than pupils with fathers working as professionals, semi-professionals or manual workers (knowledge_Professional_= 0.53±0.20 (mean + SD), knowledge_Routine non-manual worker_ = 0.45±0.22, knowledge_Semi-professional_ = 0.51±0.24, knowledge_Manual worker_ = 0.49±0.23, see Table S2 in supplement)). (see Table 2).

Table1: Results of the LMM on the influence of pupils’ age (z-transformed), gender (female or male), time (before or after TCE intervention), village category (rural, suburban or urban) and father’ jobs category (manual worker, professional, routine non-manual worker or semi-professional) on their total score (average of the scores for the knowledge, habit and behaviour sections).

| Term | Estimate | S.E. | df | t-values | p-values |
| --- | --- | --- | --- | --- | --- |
| Age | -0,00036 | 0,0064 | 305,90 | -0,056 | 0,96 |
| Gender | -0,015 | 0,0099 | 651,11 | -1,56 | 0,12 |
| Time | -0,049 | 0,0075 | 581,97 | -6,56 | p < 0.001 |
| Village Category: Suburban^a^ | -0,014 | 0,018 | 139,92 | -0,77 | 0,45 |
| Village Category:  Urban^a^ | 0,031 | 0,033 | 241,19 | 0,93 | 0,35 |
| Father’s job category: Professional^b^ | 0,027 | 0,028 | 700,96 | 0,95 | 0,34 |
| Father’s job category:  Routine non-manual worker^b^ | -0,013 | 0,013 | 644,38 | -1,0095 | 0,31 |
| Father’s job category: Semi-professional^b^ | 0,022 | 0,016 | 663,65 | 1,35 | 0,18 |

^a^ Reference level is rural

^b^ Reference level is manual worker

Table 2: Results of the CLMM on the influence of pupils’ age (z-transformed), gender (female or male), time (before or after TCE intervention), village category (rural, suburban or urban) and father’ jobs category (manual worker, professional, routine non-manual worker or semi-professional) on their score of knowledge

| Term | Estimate | S.E. | z-values | p-values |
| --- | --- | --- | --- | --- |
| Age | 0,11 | 0,097 | 1,11 | 0,27 |
| Gender | 0,041 | 0,13 | 0,32 | 0,75 |
| Time | -0,73 | 0,091 | -8,00 | p < 0.001 |
| Village category: Suburban^a^ | 0,064 | 0,32 | 0,20 | 0,84 |
| Village category: Urban^a^ | 0,20 | 0,48 | 0,41 | 0,68 |
| Father’s jobs_category:  Professional^b^ | 0,47 | 0,35 | 1,36 | 0,17 |
| Father’s jobs category:  Routine non-manual worker^b^ | -0,34 | 0,16 | -2,13 | p < 0,05 |
| Father’s jobs category:  Semi-professional^b^ | -0,0026 | 0,20 | -0,013 | 0,99 |

^a^ Reference level is rural

^b^ Reference level is manual worker

Table 3: Results of the CLMM on the influence of pupils’ age (z-transformed), gender (female or male), time (before or after TCE intervention), village category (rural, suburban or urban) and father’ jobs category (manual worker, professional, routine non-manual worker or semi-professional) on their score of behaviour

| Term | Estimate | S.E. | z-values | p-values |
| --- | --- | --- | --- | --- |
| Age | 0,13 | 0,083 | 1,59 | 0,11 |
| Gender | -0,40 | 0,15 | -2,72 | p < 0,01 |
| Time | -0,25 | 0,11 | -2,22 | p < 0,05 |
| Village category: Suburban^a^ | 0,012 | 0,22 | 0,054 | 0,96 |
| Village category: Urban^a^ | -0,087 | 0,41 | -0,21 | 0,83 |
| Father’s jobs_category:  Professional^b^ | -0,47 | 0,42 | -1,10 | 0,27 |
| Father’s jobs category:  Routine non-manual worker^b^ | 0,12 | 0,19 | 0,61 | 0,54 |
| Father’s jobs category:  Semi-professional^b^ | 0,23 | 0,24 | 0,96 | 0,34 |

^a^ Reference level is rural

^b^ Reference level is manual worker
